# Supplementary material for: Circulating Tumor DNA as a Preoperative Marker of Recurrence in Patients with Peritoneal Metastases of Colorectal Cancer: A Clinical Feasibility Study
Source: J Clin Med. 2020 Jun 4;9(6):1738. doi: 10.3390/jcm9061738 (PMC7357031; doi:10.3390/jcm9061738)
Supplement: Supplementary file 1 [file jcm-09-01738-s001.zip › Supplementary data/Figure S1.docx]

**Figure S1:** Mean cfDNA yields of plasma samples taken preoperatively and during follow-up. All samples were negative for ctDNA.
