# Supplementary material for: Circulating Tumor DNA as a Preoperative Marker of Recurrence in Patients with Peritoneal Metastases of Colorectal Cancer: A Clinical Feasibility Study
Source: J Clin Med. 2020 Jun 4;9(6):1738. doi: 10.3390/jcm9061738 (PMC7357031; doi:10.3390/jcm9061738)
Supplement: Supplementary file 1 [file jcm-09-01738-s001.zip › Supplementary data/Supplementary materials and methods.docx]

**SUPPLEMENTARY MATERIALS AND METHODS**

**Clinical and pathological data**

The following clinical and pathological data were obtained from the patient’s records: age, sex, body-mass index, ASA-classification, primary tumour location, TNM-stage and tumour histology. Information relating to prior treatment was obtained, including: previous administration of systemic chemotherapy; previous resection of the primary tumour; the presence of lymph node or haematogenous metastases at the time of CRS-HIPEC; PCI scores from zero to 39, measured by DLS.^1,2^

**Blood processing**

Preoperative blood was collected through a central venous or arterial line into two 10ml Cell-free DNA Blood Collection Tubes (Streck Inc, Nebraska, USA). If Streck tubes were unavailable, three 6ml EDTA tubes (BD Vacutainer, NJ, USA). All samples were centrifuged within eight hours of collection at 820g for 10 minutes, the plasma layer transferred to a 50ml tube without disturbing the buffy coat, then transferred to 1.5ml tubes and spun at 16 000g for 10 minutes. The plasma was subsequently transferred into 1.8ml Nunc CryoTubes (Merck KGaA, Darmstadt, Germany) without disturbing the pellet and stored at -80˚C.

### **DNA isolation and mutation analysis**

Biopsied PMs were processed according to a routine protocol in which the formalin-fixed paraffin-embedded tumour tissue was manually macro-dissected from serial sections guided by a haematoxylin and eosin stained tissue section on which the tumour region was marked by a pathologist. ^3,4^ Genomic DNA from PMs were analysed using the 48-gene, 212-amplicon TruSeq Amplicon Cancer Panel (TSACP) of commonly mutated onco- and tumour suppressor genes of solid malignancies Illumina Inc., CA, USA) as previously described. ^4^ One sample could not be successfully analysed using TSACP, so underwent testing with High Resolution Melting assay follow by Sanger sequencing (HRM-sequencing) for *KRAS* and *NRAS* exon 2-4, *BRAF* exon 15 and *PIK3CA* exon 9 and 20 as previously described. ^5^

**gBlocks and ddPCR**

All gBlocks were individually diluted to a concentration of 1% in 8ng of pooled wild-type human DNA (Megapool Reference DNA, Leica Biosystems, Wetzlar, Germany), which had been sheared to an average length of 150 base-pairs by ultrasonification (Covaris Focussed-ultrasonicator M220, MA, USA) to mimic the properties of cfDNA fragmentation. Droplet digital PCR was performed on a QX200 (BioRad) using 8ul undiluted cfDNA elution under the following thermo-cycler conditions: 95˚c for 10 minutes, 40 cycles of 94˚c for 30 seconds then 53˚c (55˚c for KRAS screening kit) for 1 minute, followed by 98˚c for 10 minutes and an overnight hold at 12˚c. Thresholds to determine positive droplets were set individually for each kit based primarily on the performance of the gBlock analysis (Supp. table 1A). Results were deemed mutant-positive if four or more FAM-dye positive droplets were observed.

**References**

1. Elias D, Gilly F, Boutitie F, et al. Peritoneal colorectal carcinomatosis treated with surgery and perioperative intraperitoneal chemotherapy: retrospective analysis of 523 patients from a multicentric French study. J Clin Oncol*.* 2010;28(1):63-68.

2. Sugarbaker PH. Intraperitoneal chemotherapy and cytoreductive surgery for the prevention and treatment of peritoneal carcinomatosis and sarcomatosis. Seminars in surgical oncology*.* 1998;14(3):254-261.

3. van Essen HF, Ylstra B. High-resolution copy number profiling by array CGH using DNA isolated from formalin-fixed, paraffin-embedded tissues. Methods in molecular biology (Clifton, NJ)*.* 2012;838:329-341.

4. Sie D, Snijders PJ, Meijer GA, et al. Performance of amplicon-based next generation DNA sequencing for diagnostic gene mutation profiling in oncopathology. Cell Oncol*.* 2014;37(5):353-361.

5. Heideman DA, Thunnissen FB, Doeleman M, et al. A panel of high resolution melting (HRM) technology-based assays with direct sequencing possibility for effective mutation screening of EGFR and K-ras genes. Cell Oncol*.* 2009;31(5):329-333.
