# Supplementary material for: Circulating Tumor DNA as a Preoperative Marker of Recurrence in Patients with Peritoneal Metastases of Colorectal Cancer: A Clinical Feasibility Study
Source: J Clin Med. 2020 Jun 4;9(6):1738. doi: 10.3390/jcm9061738 (PMC7357031; doi:10.3390/jcm9061738)
Supplement: Supplementary file 1 [file jcm-09-01738-s001.zip › Supplementary data/Table S1A.pdf]

SUPPLEMENTARY TABLE 1A: Mutations detected in peritoneal metastasis solid-tissue for each patient included in the study

Patient number	Treatment sub-group	Gene	Mutations identified	Variant Allele Frequency (%)	COSMIC ID	dbSNP ID	BioRad ddPCR kit reference	ddPCR thresholds for detection	
								Channel 1	Channel 2
L-01	open-close	KRAS	(c.35G>T; p.(Gly12Val))	19	COSM520	rs121913529	dHsaMDV2010005	2000	2000
L-02	open-close	KRAS	(c.35G>A; p.(Gly12Asp))	*HRM and seq*	COSM521	rs121913529	dHsaMDV2510596	2000	2300
L-03	CRS-HIPEC	KRAS	(c.35G>A; p.(Gly12Asp))	*HRM*	COSM521	rs121913529	dHsaMDV2510596	2000	2300
L-04	CRS-HIPEC	APC	(c.4348C>T; p.(R1450Ter))	15	COSM13127	rs121913332	dHsaMDV2510508	4000	2000
L-05	CRS-HIPEC	KRAS	(c.34G>T; p.(Gly12Cys))	42	COSM516	rs121913530	dHsaMDV2510584	4500	2000
L-05	CRS-HIPEC	TP53	(c.817C>T; p.(Arg273Cys))	42	COSM10659	rs121913343	dHsaMDV2510538	4458	2200
L-06	CRS-HIPEC	TP53	(c.422G>A; p.(Cys141Tyr))	43	COSM43708	rs587781288	dHsaMDV2516744	3000	2200
L-07	CRS-HIPEC	FBXW7	(c.1273C>T; p.(Arg425Cys))	29	COSM74637	rs149680468	dHsaMDS604541535	800	1600
L-08	CRS-HIPEC	KRAS	(c.35G>A; p.(Gly12Asp))	12	COSM521	rs121913529	dHsaMDV2510596	2000	2300
L-09	CRS-HIPEC	KRAS	(c.351A>T; p.(Lys117Asn))	16	COSM28519	rs770248150	not tested in cfDNA		
L-09	CRS-HIPEC	APC	(c.4135G>T; p.(Glu1379Ter))	21	COSM18834	rs121913326	dHsaMDS2510752	2000	2100
L-09	CRS-HIPEC	PTEN	(c.650T>A; p.(Val217Asp))	20	COSM1159818	unknown	dHsaMDS451895027	3000	1700
L-09	CRS-HIPEC	TP53	(c.818G>A; p.(Arg273His))	26	COSM10660	rs28934576	dHsaMDV2010109	3100	2800
L-10	open-close	TP53	(c.844C>T; p.(Arg282Trp))	20	COSM10704	rs28934574	dHsaMDV2516902	1461	1638
L-11	CRS-HIPEC	ATM	(c.1898+2T>G)	65	unknown	rs587782124	not tested in cfDNA		
L-11	CRS-HIPEC	KRAS	(c.35G>A; p.(Gly12Asp))	50	COSM521	rs121913529	dHsaMDV2510596	2000	2300
L-11	CRS-HIPEC	SMAD4	(c.403C>T; p.(Arg135Ter))	46	COSM14168	rs377767326	dHsaMDS2513354	4204	4833
L-12	CRS-HIPEC	BRAF	(c.1799T>A; p.(Val600Glu))	4	COSM476	rs113488022	dHsaMDV2010027	2000	2000
L-12	CRS-HIPEC	PIK3CA	(c.263G>A; p.(Arg88Gln))	3	COSM746	rs121913287	dHsaMDV2510558	4000	3400
L-13	CRS-HIPEC	KRAS	(c.35G>T; p.(Gly12Val))	47	COSM520	rs121913529	dHsaMDV2010005	2000	2000
L-13	CRS-HIPEC	APC	(c.4132C>T; p.(Q1378Ter))	46	COSM18862	rs121913329	dHsaMDV2516770	1200	1150
L-13	CRS-HIPEC	TP53	(c.586C>T; p.(Arg196Ter))	50	COSM10705	rs397516435	dHsaMDV2010121	2000	2900
L-14	CRS-HIPEC	KRAS	(c.180_181delinsAA; p.(Gln61Lys))	28	COSM87298	unknown	dHsaMDS716116395	5000	2200
L-14	CRS-HIPEC	TP53	(c.524G>A; p.(Arg175His))	28	COSM10648	rs28934578	dHsaMDV2010105	1000	1200
L-15	CRS-HIPEC	SMAD4	(c.1572G>T; p.(Trp524Cys))	57	COSM3388472	unknown	dHsaMDS589983490	2900	2600
L-15	CRS-HIPEC	KRAS	(c.38G>A; p.(Gly13Asp))	34	COSM532	rs112445441	dHsaMDV2510598	not tested	not tested
L-15	CRS-HIPEC	APC	(c.4099C>T; p.(Gln1367Ter))	43	COSM13121	rs121913328	dHsaMDS2511856	1400	1800
L-15	CRS-HIPEC	TP53	(c.701A>G; p.(Tyr234Cys))	64	COSM10725	rs587780073	dHsaMDV2516900	2500	2200
L-16	CRS-HIPEC	FBXW7	(c.1393C>T; p.(Arg465Cys))	17	COSM22932	rs867384286	dHsaMDV2510506	1400	1850
L-16	CRS-HIPEC	KRAS	(c.38G>A; p.(Gly13Asp))	16	COSM532	rs112445441	dHsaMDV2510598	not tested	not tested
L-16	CRS-HIPEC	APC	(c.4037C>A; p.(Ser1346Ter))	17	COSM19084	unknown	dHsaMDS2515998	1500	1800
L-17	CRS-HIPEC	SMAD4	(c.1082G>A; p.(Arg361His))	11	COSM14122	rs377767347	dHsaMDS2515388	4800	3400
L-18	CRS-HIPEC	BRAF	(c.1799T>A; p.(Val600Glu))	14	COSM476	rs113488022	dHsaMDV2010027	2000	2000
L-18	CRS-HIPEC	TP53	(c.586C>T; p.(Arg196Ter))	57	COSM10705	rs397516435	dHsaMDV2010121	2000	2900
L-19	CRS-HIPEC	TP53	(c.560-1G>A)	68	COSM43753	rs1202793339	dHsaMDS439299774	4100	1500
L-20	open-close	KRAS	(c.35G>A; p.(Gly12Asp))	42	COSM521	rs121913529	dHsaMDV2510596	2000	2300
L-20	open-close	TP53	(c.524G>A; p.(Arg175His))	28	COSM10648	rs28934578	dHsaMDV2010105	1000	1200
L-21	open-close	BRAF	(c.1799T>A; p.(Val600Glu))	12	COSM476	rs113488022	dHsaMDV2010027	2000	2000
L-22	CRS-HIPEC	APC	(c.2626C>T p.(Arg876Ter))	64	COSM18852	rs121913333	dHsaMDS444452080	3000	1200
L-22	CRS-HIPEC	TP53	(c.711G>A; p.(Met237Ile))	59	COSM10834	rs587782664	dHsaMDV2516928	3100	2400
L-23	CRS-HIPEC	APC	(c.2626C>T p.(Arg876Ter))	15	COSM18852	rs121913333	dHsaMDS444452082	3000	1200
L-23	CRS-HIPEC	KRAS	(c.35G>T; p.(Gly12Val))	18	COSM520	rs121913529	dHsaMDV2010005	2000	2000
L-24	CRS-HIPEC	PTEN	(c.1003C>T; p.(Arg335Ter))	71	COSM5151	rs121909231	dHsaMDS593814798	5100	4800
L-24	CRS-HIPEC	APC	(c.2626C>T p.(Arg876Ter))	38	COSM18852	rs121913333	dHsaMDS444452081	3000	1200
L-24	CRS-HIPEC	KRAS	(c.35G>T; p.(Gly12Val))	61	COSM520	rs121913529	dHsaMDV2010005	2000	2000
L-24	CRS-HIPEC	TP53	(c.524G>A; p.(Arg175His))	74	COSM10648	rs28934578	dHsaMDV2010105	1000	1200
L-25	CRS-HIPEC	KRAS	(c.35G>C; p.(Gly12Ala))	37	COSM522	rs121913529	dHsaMDV2510586	2000	2000
L-25	CRS-HIPEC	GNAS	(c.602G>A; p.(Arg201His))	12	COSM27895	rs121913495	not tested in cfDNA		
L-26	CRS-HIPEC	BRAF	(c.1799T>A; p.(Val600Glu))	14	COSM476	rs113488022	dHsaMDV2010027	2000	2000
L-27	CRS-HIPEC	KRAS	(c.35G>A; p.(Gly12Asp))	5	COSM521	rs121913529	dHsaMDV2510596	2000	2300
L-28	CRS-HIPEC	KRAS	(c.34G>T; p.(Gly12Cys))	22	COSM516	rs121913530	dHsaMDV2510584	4500	2000
L-29	CRS-HIPEC	TP53	(c.857A>T; p.(Glu286Val))	24	COSM43936	rs1057519985	dHsaMDS2516076	3600	2500
L-30	open-close	KRAS	(c.35G>A; p.(Gly12Asp)) confirmed by HRM	8	COSM521	rs121913529	dHsaMDV2510596	2000	2300
L-30	open-close	GNAS	(c.602G>A; p. (Arg201His))	9	COSM27895	rs121913495	not tested in cfDNA		

HRM: High Resolution Melt sequencing (no VAF% avaialble)
