# Supplementary material for: Circulating Tumor DNA as a Preoperative Marker of Recurrence in Patients with Peritoneal Metastases of Colorectal Cancer: A Clinical Feasibility Study
Source: J Clin Med. 2020 Jun 4;9(6):1738. doi: 10.3390/jcm9061738 (PMC7357031; doi:10.3390/jcm9061738)
Supplement: Supplementary file 1 [file jcm-09-01738-s001.zip › Supplementary data/Table S1B.pdf]

SUPPLEMENTARY TABLE 1B: ddPCR results of cfDNA samples taken pre-operatively and and after surgery (CRS-HIPEC or open-close)

			preoperative				up to 1 month after surgery#				up to 3 months after surgery#				up to 6 months after surgery#				up to 12 months after surgery#				up to 18 months after surgery#				up to 24 months after surgery#			
**Patient number**	**mutation detected in metastasis**	**ddPCR kit used**	**mutant copies/reaction**	**wild-type copies/reaction**	**vaf%**	**cfDNA input (ng)**	**mutant copies/reaction**	**wild-type copies/reaction**	**vaf%**	**cfDNA input (ng)**	**mutant copies/reaction**	**wild-type copies/reaction**	**vaf%**	**cfDNA input (ng)**	**mutant copies/reaction**	**wild-type copies/reaction**	**vaf%**	**cfDNA input (ng)**	**mutant copies/reaction**	**wild-type copies/reaction**	**vaf%**	**cfDNA input (ng)**	**mutant copies/reaction**	**wild-type copies/reaction**	**vaf%**	**cfDNA input (ng)**	**mutant copies/reaction**	**wild-type copies/reaction**	**vaf%**	**cfDNA input (ng)**
L-1	KRAS (c.35G>T; p.(Gly12Val))	KRAS G12/13 screen	0	418	0	1.4																								
L-1	KRAS (c.35G>T; p.(Gly12Val))	mutation specific	0	364	0	1.2																								
L-2	KRAS (c.35G>A; p.(Gly12Asp))	KRAS G12/13 screen	**13.2**	510	2.5	1.7																								
L-2	KRAS (c.35G>A; p.(Gly12Asp))	mutation specific	**12.6**	458	2.7	1.5																								
L-3	KRAS (c.35G>A; p.(Gly12Asp))	KRAS G12/13 screen	0	664	0	2.2	0	3700		12.2													0	750		2.47	0	918		3.02
L-3	KRAS (c.35G>A; p.(Gly12Asp))	mutation specific	0	644	0	2.1	0	4140		13.6													0	914		3.01	*1.8*	900		2.97
L-4	*No KRAS mutation*	KRAS G12/13 screen	*1.4*	550	0.3	1.8	0	2320		7.6									0	672		2.21	0	508		1.67				
L-4	APC (c.4348C>T; (p.R1450Ter))	mutation specific	0	714	0.0	2.3	0	3080		10.1									0	968		3.18	0	676		2.22				
L-5	KRAS (c.34G>T; p.(Gly12Cys))	KRAS G12/13 screen	0	420	0	1.4	0	3020		9.9									*1.4*	538		1.77								
L-5	TP53 (c.817C>T; p.(Arg273Cys))	mutation specific	*1.4*	384	0	1.3	*2.8*	3400		11.2									*1.6*	636		2.10								
L-5	KRAS (c.34G>T; p.(Gly12Cys))	mutation specific	0	400	0	1.3	0	3280		10.8									0	540		1.78								
L-6	TP53 (c.422G>A; p.(Cys141Tyr))	mutation specific	0	550	0.0	1.8	0	994		3.3					0	766		2.52												
L-6	*No KRAS mutation*	KRAS G12/13 screen	0	458	0.0	1.5	0	852		2.8					0	658		2.16												
L-7	*No KRAS mutation*	KRAS G12/13 screen	0	1216	0.0	4.0	0	1128		3.7																				
L-7	FBXW7 (c.1273C>T; (p.Arg425Cys))	mutation specific	0	1302	0.0	4.3	0	1452		4.8																				
L-8	KRAS (c.35G>A; p.(Gly12Asp))	KRAS G12/13 screen	0	400	0	1.3	*2.8*	6140		20.2					0	514		1.69												
L-8	KRAS (c.35G>A; p.(Gly12Asp))	mutation specific	0	344	0	1.1	*1.6*	6400		21.1					0	588		1.93												
L-9	*KRAS (not in screen kit)*	KRAS G12/13 screen	*1.6*	870	0.2	2.9	*1.4*	6240		20.5									0	946		3.11								
L-9	APC (c.4135G>T; p.(Glu1379Ter))	mutation specific	0	848	0	2.8	0	5780		19.0									**76**	850	8.21	3.05								
L-9	TP53 (c.818G>A; p.(Arg273His))	mutation specific	0	996	0	3.3	0	6360		20.9									**116**	966	10.72	3.56								
L-9	PTEN (c.650T>A: (p.Val217Asp))	mutation specific	0	1214	0	4.0	0	7780		25.6									**80**	1024	7.25	3.63								
L-10	*No KRAS mutation*	KRAS G12/13 screen	0	736	0	2.4																								
L-10	TP53 (c.844C>T; p.(Arg282Trp)	mutation specific	0	870	0	2.9																								
L-11	KRAS (c.35G>A; p.(Gly12Asp))	KRAS G12/13 screen	**11.4**	658	1.7	2.2	0	1934		6.4					0	652		2.14	0	646		2.13								
L-11	SMAD4 (c.403C>T; p.(Arg135Ter))	mutation specific	**9**	804	1.1	2.7	0	2440		8.0					0	890		2.93	*1.4*	784		2.58								
L-12	*No KRAS mutation*	KRAS G12/13 screen	0	968	0	3.2	*3.2*	15980		52.6	0	940		3.09	*1.6*	1350		4.45												
L-12	BRAF (c.1799T>A; p.(Val600Glu))	mutation specific	0	978	0	3.2	0	14920		49.1	0	914		3.01	0	1250		4.11												
L-12	PIK3CA (c.263G>A; (p.Arg88Gln))	mutation specific	0	1034	0	3.4	0	20160		66.3	0	1074		3.53	0	1620		5.33												
L-13	KRAS (c.35G>T; p.(Gly12Val))	KRAS G12/13 screen	**114**	938	10.8	3.5	*1.4*	2180		7.2																				
L-13	APC (c.4132C>T; p.(Gln1378Ter))	mutation specific	**108**	1068	9.2	3.9	*0*	2380		7.8																				
L-13	TP53 (c.586C>T (p.Arg196Ter))	mutation specific	**128**	1122	10.2	4.1	*1.6*	2580		8.5																				
L-13	KRAS (c.35G>T; p.(Gly12Val))	mutation specific	**74**	678	9.8	2.5	*1.6*	1720		5.7																				
L-14	KRAS (c.180_181delinsAA; p.(Gln61Lys))	KRAS G12/13 screen	0	1040	0	3.4	*3*	15540		51.1	*1.4*	1282		4.22																
L-14	TP53 (c.524G>A; p.(Arg175His))	mutation specific	0	1256	0	4.1	*3*	16220		53.4	0	1500		4.93																
L-14	KRAS (c.180_181delinsAA; p.(Gln61Lys))	mutation specific	0	1302	0	4.3	0	14660		48.2	0	1372		4.51																
L-15	KRAS (c.38G>A; p.(Gly13Asp))	KRAS G12/13 screen	0	402	0	1.3	0	2100		6.9					0	864		2.84	0	776		2.55								
L-15	APC (c.4099C>T; p.(Gln1367Ter))	mutation specific	0	632	0	2.1	*1.4*	2780		9.1					0	1208		3.97	*4.6*	940		3.11								
L-15	SMAD4 (c.1572G>T; p.(Trp524Cys))	mutation specific	0	520	0	1.7	0	2760		9.1					0	1134		3.73	0	960		3.16								
L-15	TP53 (c.701A>G; (p.Tyr234Cys))	mutation specific	0	550	0	1.8	0	2620		8.6					0	1242		4.09	0	1086		3.57								
L-16	KRAS (c.38G>A; p.(Gly13Asp))	KRAS G12/13 screen	*2.8*	860	0.3	2.8	0	1304		4.3					*1.6*	1338		4.41												
L-16	FBXW7 (c.1393C>T; p.(Arg465Cys))	mutation specific	*1.2*	846	0.1	2.8	0	1388		4.6					0	1334		4.39												
L-16	APC (c.4037C>A; p.(Ser1346Ter)	mutation specific	*3.2*	880	0.4	2.9	0	1506		5.0					0	1388		4.57												
L-17	*No KRAS mutation*	KRAS G12/13 screen	0	1244	0	4.1	0	1682		5.5	0	1340		4.41																
L-17	SMAD4 (c.1082G>A; (p.Arg361His))	mutation specific	0	1560	0	5.1	0	1740		5.7	0	1170		3.85																
L-18	*No KRAS mutation*	KRAS G12/13 screen	0	546	0	1.8	0	1524		5.0									0	2120		6.97								
L-18	TP53 (c.586C>T (p.Arg196Ter))	mutation specific	**6.6**	616	4	2.0	*1.6*	1840		6.1									**16**	2760	0.58	9.13								
L-18	BRAF V600E	mutation specific	**4.2**	458	1	1.5	0	1500		4.9									*2.6*	1842		6.07								
L-19	*No KRAS mutation*	KRAS G12/13 screen	0	438	0	1.4	*3.2*	15980		52.6									0	874		2.88								
L-19	TP53 c.560-1G>A splice variant	mutation specific	**11.2**	426	3	1.4	*5*	14720		48.4									**11.2**	914	1.210549	3.04								
L-20	KRAS (c.35G>A; p.(Gly12Asp))	KRAS G12/13 screen	**6.6**	600	1.1	2.0																								
L-20	TP53 (c.524G>A; p.(Arg175His))	mutation specific	**5.2**	732	0.7	2.4																								
L-21	*No KRAS mutation*	KRAS G12/13 screen	*1.4*	742	0.2	2.4																								
L-21	BRAF V600E	mutation specific	0	662	0.0	2.2																								
L-22	*No KRAS mutation*	KRAS G12/13 screen	0	440	0	1.4	0	838		2.8																				
L-22	APC (c.2626C>T (p.Arg876Ter))	mutation specific	0	476	0	1.6	0	1054		3.5																				
L-22	TP53 (c.711G>A (p.Met237Ile))	mutation specific	0	492	0	1.6	0	958		3.2																				
L-23	KRAS (c.35G>T; p.(G12V))	KRAS G12/13 screen	*1.6*	348	0.5	1.2	0	2580		8.5	*3.4*	5420		17.84	0	650		2.14	0	770		2.53								
L-23	APC (c.2626C>T (p.Arg876Ter))	mutation specific	*3*	412	0.7	1.4	0	3280		10.8	*3.2*	6720		22.12	0	730		2.40	0	924		3.04								
L-23	KRAS (c.35G>T; p.(G12V))	mutation specific	*1.6*	280	0.6	0.9	0	2080		6.8	*3.2*	4400		14.48	0	578		1.90	0	670		2.20								
L-24	KRAS (c.35G>T; p.(Gly12Val))	KRAS G12/13 screen	**54**	5140	1.0	17.1	*1.4*	3000		9.9					*1.4*	7920		26.06	*1.4*	2280		7.50	**42**	1848	2.22	6.22				
L-24	TP53 (c.524G>A; p.(Arg175His))	mutation specific	**34**	6380	0.5	21.1	*0*	3580		11.8					*1.6*	9620		31.65	*4.8*	2420		7.98	**32**	2200	1.43	7.34				
L-24	APC (c.2626C>T (p.Arg876Ter))	mutation specific	**28**	6040	0.5	20.0	0	3440		11.3					*1.6*	9300		30.60	*1.6*	2500		8.23	**24**	2220	1.07	7.38				
L-24	PTEN (c.1003C>T; (p.Arg335Ter))	mutation specific	**20**	5060	0.4	16.7	0	2880		9.5					0	8060		26.51	0	2100		6.91	**20**	1820	1.09	6.05				
L-25	KRAS (c.35G>C (p.Gly12Ala))	KRAS G12/13 screen	**8.6**	1182	0.7	3.9	*4.8*	29260		96.3																				
L-25	KRAS (c.35G>C (p.Gly12Ala))	mutation specific	**5.2**	1008	0.5	3.3	0	22020		72.4																				
L-26	*No KRAS mutation*	KRAS G12/13 screen	0	866	0	2.8	0	1046		3.4																				
L-26	BRAF (c.1799T>A; p.(Val600Glu))	mutation specific	**6**	936	1	3.1	0	972		3.2																				
L-27	KRAS (c.35G>A; p.(Gly12Asp))	KRAS G12/13 screen	**11.6**	534	2.1	1.8	**12.4**	3560	0.35	11.8					**24**	916	2.55	3.09												
L-28	KRAS (c.34G>T; p.(Gly12Cys))	KRAS G12/13 screen	0	912	0	3.0	0	2340		7.7					0	1840		6.05												
L-29	*No KRAS mutation*	KRAS G12/13 screen	0	474	0	1.6	0	504		1.7	0	804		2.64	0	1144		3.76												
L-29	TP53 (c.857A>T; (p.Glu286Val))	mutation specific	0	616	0	2.0	0	1520		5.0	0	1130		3.72	0	1560		5.13												
L-30	KRAS c.35G>A p.G12D	KRAS G12/13 screen	0	334	0	1.1																								
L-30	KRAS c.35G>A p.G12D	mutation specific	0	378	0	1.2																								

italics result considered negative: <4 FAM-positive droplets
bold result considered positive: >4 FAM-positive droplets
* not included in cfDNA calculation
# scheduled follow-up time-point. Actual sampling time may differ - refer to figure 4.

The KRAS ddPCR G12/13 screening kit tested the following mutations

c.35G>C p.G12A
c.34G>T p.G12C
c.35G>A p.G12D
c.34G>C p.G12R
c.34G>A p.G12S
c.35G>T p.G12V
c.38G>A p.G13D
