# Supplementary material for: Circulating Tumor DNA as a Preoperative Marker of Recurrence in Patients with Peritoneal Metastases of Colorectal Cancer: A Clinical Feasibility Study
Source: J Clin Med. 2020 Jun 4;9(6):1738. doi: 10.3390/jcm9061738 (PMC7357031; doi:10.3390/jcm9061738)
Supplement: Supplementary file 1 [file jcm-09-01738-s001.zip › Supplementary data/Table S1C.pdf]

SUPPLEMENTARY TABLE 1C: mean cfDNA yield (ct+cfDNA) in ng/ml plasma

Patient ID	CRS-HIPEC (n=24)	Open-close (n=6)	ctDNA detected	final elution volume (ul)*
L-01	-	4.8	-	58
L-02	-	6.1	yes	60
L-03	8.1	-	-	56
L-04	7.8	-	-	55
L-05	5	-	-	60
L-06	6.2	-	-	63
L-07	15.6	-	-	53
L-08	4.6	-	-	61
L-09	12.2	-	-	55
L-10	-	9.9	-	59
L-11	9.2	-	yes	60
L-12	12.3	-	-	59
L-13	13.2	-	yes	51
L-14	14.8	-	-	58
L-15	6.5	-	-	59
L-16	10.7	-	-	58
L-17	17.4	-	-	60
L-18	6.7	-	yes	53
L-19	5.5	-	yes	54
L-20	-	8.3	yes	56
L-21	-	8.7	-	52
L-22	6	-	-	60
L-23	4.3	-	-	56
L-24	70.4	-	yes	57
L-25	13.6	-	yes	53
L-26	11.2	-	yes	54
L-27	6.7	-	yes	60
L-28	11.3	-	-	52
L-29	6.7	-	-	50
L-30	-	4.4	-	60

*initial elution volume was 90ul before transfer to the final elution tubes
