# Supplementary material for: Circulating Tumor DNA as a Preoperative Marker of Recurrence in Patients with Peritoneal Metastases of Colorectal Cancer: A Clinical Feasibility Study
Source: J Clin Med. 2020 Jun 4;9(6):1738. doi: 10.3390/jcm9061738 (PMC7357031; doi:10.3390/jcm9061738)
Supplement: Supplementary file 1 [file jcm-09-01738-s001.zip › Supplementary data/Table S1D.pdf]

SUPPLEMENTARY TABLE 1D: gBlock sequences used to test ddPCR kits used in this study

APC (c.2626C>T (p.Arg876Ter))
TAGTTCTCGTTCTGAAAAAGATAGAAGTTTGGAGAGAGAACGCGGAATTGGTCTAGGCAACTACCATCCAGCAACAGAAAATCCAGGAACTTCTTCAAAG**T**GAGGTTTGCAGATCTCCACCACTGCAGCCCAGATTGCCAAAGTCATGGAAGAAGTGTCAGCCATTCATACCTCTCAGGAAGACAGAAGTTCTGGGTCTA

APC (c.4348C>T; (p.R1450Ter))
CATTATAAGCCCCAGTGATCTTCCAGATAGCCCTGGACAAACCATGCCACCAAGCAGAAGTAAAACACCTCCACCACCTCCTCAAACAGCTCAAACCAAG**T**GAGAAGTACCTAAAAATAAAGCACCTACTGCTGAAAAGAGAGAGAGTGGACCTAAGCAAGCTGCAGTAAATGCTGCAGTTCAGAGGGTCCAGGTTCTTC

APC (c.3871C>T; p.(Gln1291Ter))
CCAAGAAACAATACAGACTTATTGTGTAGAAGATACTCCAATATGTTTTTCAAGATGTAGTTCATTATCATCTTTGTCATCAGCTGAAGATGAAATAGGATGTAAT**T**AGACGACACAGGAAGCAGATTCTGCTAATACCCTGCAAATAGCAGAAATAAAAGAAAAGATTGGAACTAGGTCAGCTGAAGATCCTGTGAGCG

APC (c.4037C>A; p.(Ser1346Ter)
GGAACTAGGTCAGCTGAAGATCCTGTGAGCGAAGTTCCAGCAGTGTCACAGCACCCTAGAACCAAATCCAGCAGACTGCAGGGTTCTAGTTTATCTTCAGAAT**A**AGCCAGGCACAAAGCTGTTGAATTTTCTTCAGGAGCGAAATCTCCCTCCAAAAGTGGTGCTCAGACACCCAAAAGTCCACCTGAACACTATGTTCA

APC (c.4099C>T; p.(Gln1367Ter))
AATCCAGCAGACTGCAGGGTTCTAGTTTATCTTCAGAATCAGCCAGGCACAAAGCTGTTGAATTTTCTTCAGGAGCGAAATCTCCCTCCAAAAGTGGTGCT**T**AGACACCCAAAAGTCCACCTGAACACTATGTTCAGGAGACCCCACTCATGTTTAGCAGATGTACTTCTGTCAGTTCACTTGATAGTTTTGAGAGTCGTTCGA

APC (c.4132C>T; p.(Q1378Ter))
AGAATCAGCCAGGCACAAAGCTGTTGAATTTTCTTCAGGAGCGAAATCTCCCTCCAAAAGTGGTGCTCAGACACCCAAAAGTCCACCTGAACACTATGTT**T**AGGAGACCCCACTCATGTTTAGCAGATGTACTTCTGTCAGTTCACTTGATAGTTTTGAGAGTCGTTCGATTGCCAGCTCCGTTCAGAGTGAACCATGCA

APC (c.4135G>T; p.(Glu1379Ter))
ATCAGCCAGGCACAAAGCTGTTGAATTTTCTTCAGGAGCGAAATCT**C**CCTCCAAAAGTGGTGCTCAGACACCCAAAAGTCCACCTGAACACTATGTTCAGTAGACCCCACTCATGTTTAGCAGATGTACTTCTGTCAGTTCACTTGATAGTTTTGAGAGTCGTTCGATTGCCAGCTCCGTTCAGAGTGAACCATGCAGTG

BRAF (c.1799T>A; p.(Val600Glu))
GGAAAATGAGATCTACTGTTTTCCTTTACTTACTACACCTCAGATATATTTCTTCATGAAGACCTCACAGTAAAAATAGGTGATTTTGGTCTAGCTACAG**A**GAAATCTCGATGGAGTGGGTCCCATCAGTTTGAACAGTTGTCTGGATCCATTTTGTGGATGGTAAGAATTGAGGCTATTTTTCCACTGATTAAATTTTT

FBXW7 (c.1393C>T; p.(Arg465Cys))
CAACATCATCATTAGTGGATCTACAGATCGGACACTCAAAGTGTGGAATGCAGAGACTGGAGAATGTATACACACCTTATATGGGCATACTTCCACTGTG**T**GTTGTATGCATCTTCATGAAAAAAGGTAAGGGAAAATCTTGTCATGGTTGGGACTTCTTCCTCTATTATTGGTGAATTTGGGCCCTTTCTGTGTAGAGT

FBXW7 (c.1273C>T; (p.Arg425Cys))
TGCAGAGTTGTTAGCGGTTCTCGAGATGCCACTCTTAGGGTTTGGGATATTGAGACAGGCCAGTGTTTACATGTTTTGATGGGTCATGTTGCAGCAGTC**T**GCTGTGTTCAATATGATGGCAGGAGGGTTGTTAGTGGAGCATATGATTTTATGGTAAAGGTGTGGGATCCAGAGACTGAAACCTGTCTACACACGTTGCA

GNAS (c.602G>A; (p.Arg201His))
CTACTCCAGACCTTTGCTTTAGATTGGCAATTATTACTGTTTCGGTTGGCTTTGGTGAGATCCATTGACCTCAATTTTGTTTCAGGACCTGCTTCGCTGCC**A**TGTCCTGACTTCTGGAATCTTTGAGACCAAGTTCCAGGTGGACAAAGTCAACTTCCAGTAAGCCAACTGTTACCTTTTTATATAACAGAGATCATGGT

KRAS (c.35G>A; p.(Gly12Asp))
GTGTGACATGTTCTAATATAGTCACATTTTCATTATTTTTATTATAAGGCCTGCTGAAAATGACTGAATATAAACTTGTGGTAGTTGGAGCTG**A**TGGCGTAGGCAAGAGTGCCTTGACGATACAGCTAATTCAGAATCATTTTGTGGACGAATATGATCCAACAATAGAGGTAAATCTTGTTTTAATATGC

KRAS (c.35G>T; p.(Gly12Val))
GTGTGACATGTTCTAATATAGTCACATTTTCATTATTTTTATTATAAGGCCTGCTGAAAATGACTGAATATAAACTTGTGGTAGTTGGAGCTG**T**TGGCGTAGGCAAGAGTGCCTTGACGATACAGCTAATTCAGAATCATTTTGTGGACGAATATGATCCAACAATAGAGGTAAATCTTGTTTTAATATGC

KRAS (c.38G>A; p.(Gly13Asp))
GTGTGACATGTTCTAATATAGTCACATTTTCATTATTTTTATTATAAGGCCTGCTGAAAATGACTGAATATAAACTTGTGGTAGTTGGAGCTGGTG**A**CGTAGGCAAGAGTGCCTTGACGATACAGCTAATTCAGAATCATTTTGTGGACGAATATGATCCAACAATAGAGGTAAATCTTGTTTTAATATGC

KRAS (c.34G>T; p.(Gly12Cys))
GTGTGACATGTTCTAATATAGTCACATTTTCATTATTTTTATTATAAGGCCTGCTGAAAATGACTGAATATAAACTTGTGGTAGTTGGAGCT**T**GTGGCGTAGGCAAGAGTGCCTTGACGATACAGCTAATTCAGAATCATTTTGTGGACGAATATGATCCAACAATAGAGGTAAATCTTGTTTTAATATGC

KRAS (c.180_181delinsAA; p.(Gln61Lys))
GTAATAATCCAGACTGTGTTTCTCCCTTCTCAGGATTCCTACAGGAAGCAAGTAGTAATTGATGGAGAAACCTGTCTCTTGGATATTCTCGACACAGCAGG**AA**AAGAGGAGTACAGTGCAATGAGGGACCAGTACATGAGGACTGGGGAGGGCTTTCTTTGTGTATTTGCCATAAATAATACTAAATCATTTGAAGATAT

KRAS (c.351A>T; p.(Lys117Asn))
GTTACTAATGACTGTGCTATAACTTTTTTTTCTTTCCCAGAGAACAAATTAAAAGAGTTAAGGACTCTGAAGATGTACCTATGGTCCTAGTAGGAAATAA**T**TGTGATTTGCCTTCTAGAACAGTAGACACAAAACAGGCTCAGGACTTAGCAAGAAGTTATGGAATTCCTTTTATTGAAACATCAGCAAAGACAAGACAG

KRAS c.35G>C (p.Gly12Ala))
GTGTGACATGTTCTAATATAGTCACATTTTCATTATTTTTATTATAAGGCCTGCTGAAAATGACTGAATATAAACTTGTGGTAGTTGGAGCTG**C**TGGCGTAGGCAAGAGTGCCTTGACGATACAGCTAATTCAGAATCATTTTGTGGACGAATATGATCCAACAATAGAGGTAAATCTTGTTTTAATATGC

MET (c.3029C>T (p.Thr1010Ile))
TCTCTGTTTTAAGATCTGGGCAGTGAATTAGTTCGCTACGATGCAAGAGTACACACTCCTCATTTGGATAGGCTTGTAAGTGCCCGAAGTGTAAGCCCAA**T**TACAGAAATGGTTTCAAATGAATCTGTAGACTACCGAGCTACTTTTCCAGAAGGTATATTTCAGTTTATTGTTCTGAGAAATACCTATACATATACCTC

PIK3CA (c.263G>A; (p.Arg88Gln))
AATACCCCCTCCATCAACTTCTTCAAGATGAATCTTCTTACATTTTCGTAAGTGTTACTCAAGAAGCAGAAAGGGAAGAATTTTTTGATGAAACAAGAC**A**ACTTTGTGACCTTCGGCTTTTTCAACCCTTTTTAAAAGTAATTGAACCAGTAGGCAACCGTGAAGAAAAGATCCTCAATCGAGAAATTGGTATGATACAA

PIK3CA (c.3019G>C; (p.G1007R))
GTTCTTATTACTTATAGGTTTCAGGAGATGTGTTACAAGGCTTATCTAGCTATTCGACAGCATGCCAATCTCTTCATAAATCTTTTCTCAATGATGCTT**C**GCTCTGGAATGCCAGAACTACAATCTTTTGATGACATTGCATACATTCGAAAGACCCTAGCCTTAGATAAAACTGAGCAAGAGGCTTTGGAGTATTTCA

PIK3CA (c.325_327delGAA; p.(Glu109del))
AGAAGCAGAAAGGGAAGAATTTTTTGATGAAACAAGACGACTTTGTGACCTTCGGCTTTTTCAACCCTTTTTAAAAGTAATTGAACCAGTAGGCAACCGTGAAAAGATCCTCAATCGAGAAATTGGTATGATACAATATCCTATTCTAAAATGCAAATAACCATAAAGCTTAACTGTTGTCCCTTTCTAAAATATTTCTG

PTEN (c.1003C>T (p.Arg335Ter))
TAGCATTTGCAGTATAGAGCGTGCAGATAATGACAAGGAATATCTAGTACTTACTTTAACAAAAAATGATCTTGACAAAGCAAATAAAGACAAAGCCAAC**T**GATACTTTTCTCCAAATTTTAAGGTCAGTTAAATTAAACATTTTGTGGGGGTTGTTGACTTGTATGTATGTGATGTGTGTTTAATTCTAGGAGTACAG

PTEN (c.650T>A; (p.Val217Asp))

TATTAATCATTAAAATCGTTTTTGACAGTTTGACAGTTAAAGGCATTTCCTGTGAAATAATACTGGTATGTATTTAACCATGCAGATCCTCAGTTTGTGG**A**CTGCCAGCTAAAGGTGAAGATATATTCCTCCAATTCAGGACCCACACGACGGGAAGACAAGTTCATGTACTTTGAGTTCCCTCAGCCGTTACCTGTGTG

SMAD4 (c.403C>T ;p.(Arg135Ter))

GGCCTGATCTTCACAAAAATGAACTAAAACATGTTAAATATTGTCAGTATGCGTTTGACTTAAAATGTGATAGTGTCTGTGTGAATCCATATCACTACGAA**T**GAGTTGTATCACCTGGAATTGGTAAGTAGACTTTGCTTTCATCCTAAGAAACATAAAGGGAAAAGGATCTCAATAGTGTTTCAATTTTTGTAAGTTAA

SMAD4 (c.1082G>A; (p.Arg361His))

ACTTTGAAATGGATGTTCAGGTAGGAGAGACATTTAAGGTTCCTTCAAGCTGCCCTATTGTTACTGTTGATGGATACGTGGACCCTTCTGGAGGAGATC**A**CTTTTGTTTGGGTCAACTCTCCAATGTCCACAGGACAGAAGCCATTGAGAGAGCAAGGTATTGATTGTATAGTCAGATAGTTACTTTAAAAAATTGAGCA

SMAD4 (c.1572G>T; p.(Trp524Cys))

GTGTTGATGACCTTCGTCGCTTATGCATACTCAGGATGAGTTTTGTGAAAGGCTGGGGACCGGATTACCCAAGACAGAGCATCAAAGAAACACCTTGCTG**T**ATTGAAATTCACTTACACCGGGCCCTCCAGCTCCTAGACGAAGTACTTCATACCATGCCGATTGCAGACCCACAACCTTTAGACTGAGGTCTTTTACCG

TP53 (c.524G>A; p.(Arg175His))

TTTTGCCAACTGGCCAAGACCTGCCCTGTGCAGCTGTGGGTTGATTCCACACCCCCGCCCGGCACCCGCGTCCGCGCCATGGCCATCTACAAGCAGTCACAGCACATGACGGAGGTTGTGAGGC**A**CTGCCCCCACCATGAGCGCTGCTCAGATAGCGATGGTGAGCAGCTGGGGCTGGAGAGACGACAGGGCTGGTTGCCCAGGGTCCCCAGGCCTCTGATTCCTCACTGATT

TP53 (c.586C>T (p.Arg196Ter))

GCTGGGGCTGGAGAGACGACAGGGCTGGTTGCCCAGGGTCCCCAGGCCTCTGATTCCTCACTGATTGCTCTTAGGTCTGGCCCCTCCTCAGCATCTTATC**T**GAGTGGAAGGAAATTTGCGTGTGGAGTATTTGGATGACAGAAACACTTTTCGACATAGTGTGGTGGTGCCCTATGAGCCGCCTGAGGTCTGGTTTGCAA

TP53 (c.817C>T; p.(Arg273Cys))

TAAATGGGACAGGTAGGACCTGATTTCCTTACTGCCTCTTGCTTCTCTTTTCCTATCCTGAGTAGTGGTAATCTACTGGGACGGAACAGCTTTGAGGTG**T**GTGTTTGTGCCTGTCCTGGGAGAGACCGGCGCACAGAGGAAGAGAATCTCCGCAAGAAAGGGGAGCCTCACCACGAGCTGCCCCCAGGGAGCACTAAGCG

TP53 (c.818G>A; p.(Arg273His))

TAAATGGGACAGGTAGGACCTGATTTCCTTACTGCCTCTTGCTTCTCTTTTCCTATCCTGAGTAGTGGTAATCTACTGGGACGGAACAGCTTTGAGGTGC**A**TGTTTGTGCCTGTCCTGGGAGAGACCGGCGCACAGAGGAAGAGAATCTCCGCAAGAAAGGGGAGCCTCACCACGAGCTGCCCCCAGGGAGCACTAAGCG

TP53 (c.844C>T; p.(Arg282Trp)

CTTACTGCCTCTTGCTTCTCTTTTCCTATCCTGAGTAGTGGTAATCTACTGGGACGGAACAGCTTTGAGGTGCGTGTTTGTGCCTGTCCTGGGAGAGAC**T**GGCGCACAGAGGAAGAGAATCTCCGCAAGAAAGGGGAGCCTCACCACGAGCTGCCCCCAGGGAGCACTAAGCGAGGTAAGCAAGCAGGACAAGAAGCGGT

TP53 (c.422G>A; p.(Cys141Tyr))

TATCTGTTCACTTGTGCCCTGACTTTCAACTCTGTCTCCTTCCTCTTCCTACAGTACTCCCCTGCCCTCAACAAGATGTTTTGCCAACTGGCCAAGACCT**A**CCCTGTGCAGCTGTGGGTTGATTCCACACCCCCGCCCGGCACCCGCGTCCGCGCCATGGCCATCTACAAGCAGTCACAGCACATGACGGAGGTTGTGAG

TP53 (c.701A>G; (p.Tyr234Cys))

AAGGCCTCCCCTGCTTGCCACAGGTCTCCCCAAGGCGCACTGGCCTCATCTTGGGCCTGTGTTATCTCCTAGGTTGGCTCTGACTGTACCACCATCCACT**G**CAACTACATGTGTAACAGTTCCTGCATGGGCGGCATGAACCGGAGGCCCATCCTCACCATCATCACACTGGAAGACTCCAGGTCAGGAGCCACTTGCCA

TP53 (c.711G>A (p.Met237Ile))

CTGCTTGCCACAGGTCTCCCCAAGGCGCACTGGCCTCATCTTGGGCCTGTGTTATCTCCTAGGTTGGCTCTGACTGTACCACCATCCACTACAACTACAT**A**TGTAACAGTTCCTGCATGGGCGGCATGAACCGGAGGCCCATCCTCACCATCATCACACTGGAAGACTCCAGGTCAGGAGCCACTTGCCACCCTGCACAC

TP53 (c.857A>T; (p.Glu286Val))

GCTTCTCTTTTCCTATCCTGAGTAGTGGTAATCTACTGGGACGGAACAGCTTTGAGGTGCGTGTTTGTGCCTGTCCTGGGAGAGACCGGCGCACAGAGG**T**AGAGAATCTCCGCAAGAAAGGGGAGCCTCACCACGAGCTGCCCCCAGGGAGCACTAAGCGAGGTAAGCAAGCAGGACAAGAAGCGGTGGAGGAGACCAA

TP53 c.560-1G>A splice variant

GCGCTGCTCAGATAGCGATGGTGAGCAGCTGGGGCTGGAGAGACGACAGGGCTGGTTGCCCAGGGTCCCCAGGCCTCTGATTCCTCACTGATTGCTCTTA**A**GTCTGGCCCCTCCTCAGCATCTTATCCGAGTGGAAGGAAATTTGCGTGTGGAGTATTTGGATGACAGAAACACTTTTCGACATAGTGTGGTGGTGCCC
