# Supplementary material for: Circulating Tumor DNA as a Preoperative Marker of Recurrence in Patients with Peritoneal Metastases of Colorectal Cancer: A Clinical Feasibility Study
Source: J Clin Med. 2020 Jun 4;9(6):1738. doi: 10.3390/jcm9061738 (PMC7357031; doi:10.3390/jcm9061738)
Supplement: Supplementary file 1 [file jcm-09-01738-s001.zip › Supplementary data/Table S2.docx]

Table S2

Correlations between preoperative cfDNA detection (positive versus negative for ctDNA) and clinical and pathological variables. * Mann-Whitney U test. ** Fisher’s exact test. BMI: Body Mass Index. PCI: Peritoneal Cancer Index.

|  |  |  |  |  |
| --- | --- | --- | --- | --- |
|  |  |  |  |  |
|  |  | **Preoperative ctDNA analysis** | | |
| **Characteristic** |  | **ctDNA pos**  **(n=10)** | **ctDNA neg**  **(n=20)** | **p-value** |
|  |  | **Mean (SD)** | **Mean (SD)** |  |
| **BMI** |  | 26.8 (3.9) | 27.3 (5.8) | 0.801* |
| **PCI** |  | 10.1 (8.7) | 10.2 (8.2) | 0.910* |
|  |  | **n (%)** | **n (%)** |  |
| **Primary tumour location** | **Colon** | 10 (100) | 17 (85) | 0.532** |
|  | **Rectum** | 0 (0) | 3 (15) |  |
| **Differentiation grade primary** | **Good/moderate** | 9 (100) | 14 (88) | 0.520** |
|  | **Poor/signet cell** | 0 (0) | 2 (13) |  |
| **Mucinous tumour** | **Yes** | 2 (20) | 6 (30) | 0.682** |
|  | **No** | 8 (80) | 14 (70) |  |
| **Previous chemotherapy** | **Yes** | 4 (40) | 7 (35) | 1.000** |
|  | **No** | 6 (60) | 13 (65) |  |
| **Primary tumour *in situ*** | **Yes** | 3 (30) | 5 (25) | 1.000** |
| **at CRS-HIPEC** | **No** | 7 (70) | 15 (75) |  |
| **Lymph node metastases** | **Yes** | 3 (30) | 4 (21) | 0.665** |
| **at CRS-HIPEC** | **No** | 7 (70) | 15 (79) |  |
| **Liver metastases** | **Yes** | 2 (20) | 1 (5) | 0.251** |
| **at CRS-HIPEC** | **No** | 8 (80) | 19 (95) |  |
| **Procedure** | **CRS-HIPEC** | 8 (80) | 16 (80) | 1.000** |
|  | **Open-close** | 2 (20) | 4 (20) |  |
