# Supplementary material for: Circulating Tumor DNA as a Preoperative Marker of Recurrence in Patients with Peritoneal Metastases of Colorectal Cancer: A Clinical Feasibility Study
Source: J Clin Med. 2020 Jun 4;9(6):1738. doi: 10.3390/jcm9061738 (PMC7357031; doi:10.3390/jcm9061738)
Supplement: Supplementary file 1 [file jcm-09-01738-s001.zip › Supplementary data/Table S3.docx]

## Table S3

|  |  |  |  |  |
| --- | --- | --- | --- | --- |
| Correlations between clinical and pathological characteristics and Disease Free Survival (DFS) in months. *log-rank test. NR: Not Reached. BMI: Body Mass Index. ASA: American Society of Anesthesiologists Physical Status Classification System. PCI: Peritoneal Cancer Index | | | | |
|  |  | **Disease-free survival** | | |
| **Characteristic** |  | **Number of patients** | **Median DFS (95% CI)** | **p-value*** |
| **General** |  |  |  |  |
| **Age in years** | **<66** | 12 | 7.0 (0.2-13.8) | 0.122 |
|  | **≥66** | 12 | NR |  |
| **Gender** | **Male** | 14 | 7.0 (5.2-8.8) | 0.282 |
|  | **Female** | 10 | 13.0 (9.2-16.8) |  |
| **BMI** | **≤27** | 13 | 11.0 (4.2-17.8) | 0.359 |
|  | **>27** | 11 | NR |  |
| **ASA** | **I-II** | 17 | 12.0 (4.6-19.4) | 0.923 |
|  | **III** | 7 | 10.0 (2.3-17.7) |  |
| **Preoperative ctDNA** | **Yes** | 8 | 6.0 (1.8-10.2) | 0.016 |
|  | **No** | 16 | NR |  |
| **Primary tumour** |  |  |  |  |
| **T-stage** | **T1-3** | 9 | NR | 0.056 |
|  | **T4** | 15 | 7.0 (2.2-11.8) |  |
| **N-stage** | **N0** | 8 | NR | 0.594 |
|  | **N1-2** | 16 | 11.0 (3.1-18.9) |  |
| **Differentiation grade** | **Good/moderate** | 19 | 13.0 (-) | 0.051 |
|  | **Poor/signet cell** | 2 | 4.0 (-) |  |
| **Angio-invasion** | **Yes** | 8 | 7.0 (1.5-12.5) | 0.632 |
|  | **No** | 14 | 10.0 (2.6-17.4) |  |
| **Lymphatic invasion** | **Yes** | 5 | 6.0 (3.9-8.1) | 0.218 |
|  | **No** | 17 | 12.0 (8.8-15.2) |  |
| **Mucinous tumour** | **Yes** | 6 | 7.0 (1.0-13.0) | 0.243 |
|  | **No** | 18 | 13.0 (-) |  |
| **Treatment** |  |  |  |  |
| **Primary tumour *in situ* at CRS-HIPEC** | **Yes** | 7 | 7.0 (3.2-10.8) | 0.266 |
|  | **No** | 17 | 13.0 (7.9-18.1) |  |
| **PCI** | **≤10** | 16 | NR | 0.035 |
|  | **>10** | 8 | 5.0 (0.8-9.2) |  |
| **Liver metastases** | **Yes** | 2 | 2.0 (-) | <0.001 |
|  | **No** | 22 | 12.0 (8.2-15.8) |  |
| **Lymph node metastases at CRS-HIPEC** | **Yes** | 6 | 7.0 (0.3-13.7) | 0.407 |
|  | **No** | 18 | 12.0 (6.9-17.1) |  |
